# Interactive Effects of Nutrients and *Bradyrhizobium japonicum* on the Growth and Root Architecture of Soybean (*Glycine max* L.)

**DOI:** 10.3389/fmicb.2018.01000

**Published:** 2018-05-23

**Authors:** Dilfuza Egamberdieva, Dilfuza Jabborova, Stephan J. Wirth, Pravej Alam, Mohammed N. Alyemeni, Parvaiz Ahmad

**Affiliations:** ^1^Leibniz Centre for Agricultural Landscape Research (ZALF), Müncheberg, Germany; ^2^Institute of Genetics and Plant Experimental Biology, Academy of Sciences of Uzbekistan, Tashkent, Uzbekistan; ^3^Biology Department, College of Science and Humanities, Prince Sattam bin Abdulaziz University, Alkharj, Saudi Arabia; ^4^Department of Botany and Microbiology, Faculty of Science, King Saud University, Riyadh, Saudi Arabia; ^5^Department of Botany, S.P. College, Srinagar, India

**Keywords:** root system, rhizobia, nodulation, nutrient interactions, legume

## Abstract

Understanding the symbiotic performance of rhizobia and responses of plant root systems to mineral nutrient supply will facilitate the development of strategies to enhance effective rhizobia-legume symbioses. Interactive effect of nitrogen (N), phosphorus (P), and magnesium (Mg) on the symbiotic performance of soybean (*Glycine max* L.) with *Bradyrhizobium japonicum*, nodulation, root architecture, and the N concentration in plant tissue under hydroponic conditions were studied. Plant growth was significantly higher under a high N supply combined with Mg (HNHMg) than in combination with P (HNHP), which was attributed to the interaction between N and Mg ions. The plants grown at a low N concentration combined with either high or low P or Mg (LNHP, LNHMg, LNLP, and LNLMg) showed a higher nodule dry weight compared to those grown under a high N supply. We observed that the N content in the roots and shoots of soybean plants was significantly lower under LNHP or LNLP, but it was higher under HNHMg or LNHMg, indicating that Mg promotes N acquisition by the plant tissues. Neither root nor shoot growth responded significantly to P availability regardless of the N supply. We observed significant positive relationships between the number of nodules, the N content in plant tissues and the root system architecture of soybean plants grown with a variable supply of Mg combined with N, which highlights the importance of N and Mg availability in the growth medium in regulating root system architecture and nodule formation. The number of rhizobial cells colonizing soybean roots was highest under the HNHMg treatment (6.78 × 10^4^ CFUs/cm of root tip), followed by the HNLMg (4.72 × 10^4^ CFUs/cm of root tip) and LNHMg (4.10 × 10^4^ CFU/cm of root tip) treatments, and lowest under the LNLMg (1.84 × 10^4^ CFUs/cm of root tip) nutrient conditions. The results of this study contribute to new insights for the improvement of the root system and the symbiotic performance of rhizobia inoculated on legumes, stressing the importance of a balanced supply of nutrients.

## Introduction

Nutrient interactions are important in plant nutrition, impacting processes of absorption, transport, and distribution and the utilization of nutrients (Fageria, 2001; Niu et al., 2015; Li et al., 2016). Such interactions may be positive or negative, affecting plant physiological and developmental processes (Niu et al., 2015). The interaction between legumes and rhizobia, which form symbiotic associations, is especially sensitive to several factors, notably nutrient levels in growth media (Rinaudi et al., 2006; Lau et al., 2012). The effects of nutrients present in growth media on *Rhizobium*-legume symbioses and nitrogen fixation have been reported (Olivera et al., 2004; Chaudhary et al., 2008). Rhizobia require adequate mineral nutrients, such as carbon, nitrogen, phosphorus, potassium, magnesium, and several microelements for the metabolism involved in invading and successfully colonizing the host root system in order to establish an effective legume-plant host symbiosis (Paliya et al., 2014; Egamberdieva et al., 2016, 2017; Chen et al., 2017). In the rhizosphere, high competition for nutrients and niches occurs among various kinds of organisms, including the indigenous microflora, and rhizobia colonization depends on nutrient availability (Kuzyakov and Xu, 2013). An unlimited supply of essential mineral nutrients promotes plant growth improvement, successful nodule formation, and adequate N content in the plant tissues of leguminous plants due to bacterial nitrogen fixation. Therefore, mineral nutrients play a crucial role in rhizobia-host symbiotic interactions.

Magnesium (Mg), phosphorus (P), and nitrogen (N) are essential nutrient elements in plant growth and development and are involved in photosynthesis, nucleic acid metabolism, protein synthesis, enzyme activity, carbohydrate metabolism, and nitrogen fixation (Qin et al., 2012; Niste et al., 2014; Chen et al., 2017). The conversion of atmospheric N into ammonium (NH_4_), which is available to plants through the intervention of rhizobia, requires P as an essential component (Dakora and Keya, 1997). The availability of P increases nodule development and N fixation in common bean (*Phaseolus vulgaris*; Liao et al., 2004; Beebe et al., 2006; Tajini et al., 2011), soybean (*Glycine max*; Ao et al., 2010), and barrel medic (*Medicago truncatula*; Sulieman et al., 2013). On the other hand, P deficiency inhibits root and shoot growth, nodule formation, nitrogen fixation, and nitrogen acquisition (Valverde et al., 2002; Egamberdiyeva et al., 2004; Win et al., 2010). Additionally, it has been observed that Mg plays a vital role in host-rhizobia interactions, as it seems to relate to nodule formation and nitrogen fixation (Verbruggen and Hermans, 2013). Moreover, Mg is an essential element involved in the formation of chlorophyll and the photosynthetic process, acting as an allosteric enzyme regulator or cofactor; it is also known to intervene in the structural stabilization of macromolecules, such as proteins and cell tissues (Cowan, 2002; Gransee and Führs, 2013; Niu et al., 2015). Magnesium deficiencies cause a reduced content of photosynthetic pigments and reduced net CO_2_ assimilation (Cakmak and Yazici, 2010).

Nitrogen plays a central role in plant physiological processes, especially enzyme activities (Hokmalipour and Darbandi, 2011; Shiri-Janagard et al., 2012). The concentration of N is considered to be a critical factor in the beneficial association of legumes with rhizobia, and higher amounts of nitrogen result in adverse effects on plant physiology, especially in legumes (Zhou et al., 2006). Barbulova et al. (2007) observed inhibition of the symbiotic performance of rhizobia and nitrogen fixation through an increased N supply. According to Gibson and Harper (1985), inhibition of nodule formation likely allows the plant to balance substantial amounts of fixed nitrogen with plant nitrogen needs. In an earlier report, Abdel Wahab and Abd-Alla (1995) observed an increased growth, nodulation, and N fixation in soybean under low N availability in soil. In another study, increased concentration of ammonium (>5 mM) inhibited nodulation, whereas low concentration (0.4 mM) stimulated an Acacia species, growing in aeroponic culture (Weber et al., 2007).

Leidi and Rodríguez-Navarro (2000) studied the interactive effect of N and P on the growth and nodulation of common bean (*P. vulgaris*) and observed increased nodule formation and N fixation under low N and high P supply. However, relatively few studies on the interactions among N, P, and Mg on plant growth have been reported.

Currently, knowledge on the interactive effects of nutrients on rhizobia-legume symbioses and evidence of such interactions are still limited. In particular, studies on how mineral nutrient interactions affect rhizobia-legume symbioses and root system responses have not been sufficiently documented. Understanding these interactions should facilitate the development of strategies to enhance effective rhizobia-legume symbioses.

Soybean (*G. max* L. Merr.) is the most important source of oil and protein in many countries around the world (Ruiz-Sainz et al., 2005; Qin et al., 2011). Soybean requires an adequate amount of nutrients for optimal growth and development to maximize yield. Therefore, our study aimed to investigate the interactive effects of nitrogen (N) and phosphorus (P) or magnesium (Mg) on the symbiotic performance of soybean (*G. max* L.) with *Bradyrhizobium japonicum* and soybean root architecture under hydroponic conditions.

## Materials and methods

### Bacterial strain and culture conditions

Strain *B. japonicum* USDA110 was obtained from South China Agricultural University, Guangzhou, China, and was maintained on yeast extract mannitol (YEM) agar, as proposed by Vincent (1970), which had the following composition: Mannitol−10.0 g/l, K_2_HPO_4_−0.5 g/l, MgSO_4_·7H_2_O−0.2 g/l, NaCl−0.1 g/l, yeast extract−1.0 g/l, and agar−15 g/L, until use. For the preparation of the inoculant, the strain was grown on YEM medium for 5 days, and the cell concentration was adjusted to 1 × 10^8^ CFU ml^−1^ for the inoculation of soybean seedlings.

### Plant growth conditions

A hydroponic growth system experiment was conducted to assess the effects of different concentrations of N, P, and Mg on the growth of soybean inoculated with *B. japonicum*. The soybean (*G. max*) genotype YC03-3 was surface sterilized with 10% v/v NaOCl and 70% ethanol and then rinsed five times with sterile distilled water. Next, the seeds were placed on paper tissues soaked in 0.5 mM CaSO_4_ and stored in a plant growth chamber for 6 days at 26°C. The seedlings were then carefully collected and dipped in the bacterial suspension of *B. japonicum* for 5 min before being transplanted in 2-L hydroponic plastic pots filled with a modified Hoagland nutrient solution (Hoagland and Arnon, 1950; Liao et al., 2016) contained the following macronutrients (in mM): Ca(No_3_)_2_x4H_2_O−2.0; KNO_3_−3.0; K_2_SO_4_−0.5, and other nutrients (μM) Ca (HPO_4_)−50; MgCl_2_−40; Fe-EDTA−12.5; H_3_BO_3_−50; FeSO_4_−25; MnSO_4_−50; ZnSO_4_x7H_2_O−15; CuSO_4_−0.05; KCl−10; Na_2_MoO_3_−0.5; Na_2_-EDTA−25; CoCl_2_x6H_2_O−0.15; and NiSO_4_x6H_2_O−0.2. The Hoagland solution was supplemented with different levels of N, P and Mg: high P (HP) (250 μm KH_2_PO_4_) and low P (LP) (50 μmol KH_2_PO_4_), high N (HN) (3,000 μmol NH_4_NO_3_) and low N (LN) (300 μmol NH_4_NO_3_), and high Mg (HMg) (750 μmol MgSO_4_) and low Mg (LMg) (250 μmol MgSO_4_). The plants were grown under eight different nutrient solutions: (i) HNHP, (ii) HNHMg, (iii) HNLP, (iv) HNLMg, (v) LNHP, (vi) LNHMg, (vii) LNLP, and (viii) LNLMg (Table 1). The plants were grown in a greenhouse with a natural photoperiod (12 h) for 45 days (from 5 of May to 20 June) under light intensity of 500–1,800 μmol m^−2^ s^−1^. The relative humidity in the greenhouse was maintained at 65/75% day/night, and the temperature was 26/20°C day/night. The nutrient solutions were renewed twice a week. After 45 days, the plants together with their root systems were removed from the pots for the measurement of root and shoot biomass, the analysis of root system architecture, and the determination of nodule number and chlorophyll and N contents.

**Table 1:** Addition of N, P, and Mg salts to Hoagland's nutrient solution used in different treatments for the plant growth experiment in a hydroponic system.

Treatments^*^	**Nutrient concentrations**, μ**mol/L**		
	**NH_4_NO_3_**	**KH_2_PO_4_**	**MgSO_4_**
HNHP (high N and high P)	3,000	250	0
HNHMg (high N and high Mg)	3,000	0	1,000
HNLP (high N and low P)	3,000	50	0
HNLMg (high N and low Mg)	3,000	0	250
LNHP (low N and high P)	300	250	0
LNHMg (low N and high Mg)	300	0	1,000
LNLP (low N and low P)	300	50	0
LNLMg (low N and low Mg)	300	0	250

* *Hoagland nutrient solution contained the following nutrients: (in mM): Ca(No_3_)_2_x4H_2_O−2.0; KNO_3_−3.0; K_2_SO_4_−0.5; and (in μM) Ca(HPO_4_)−50; MgCl_2_−40; Fe-EDTA−12.5; H_3_BO_3_−50; FeSO_4_−25; MnSO_4_−50; ZnSO_4_x7H_2_O−15; CuSO_4_−0.05; KCl−10; Na_2_MoO_3_−0.5; Na_2_-EDTA−25; CoCl_2_x6H_2_O−0.15; and NiSO_4_x6H_2_O−0.2 (Hoagland and Arnon, 1950; Liao et al., 2016)*.

### Root morphological and architectural traits

The roots were separated from the shoots and washed carefully with water. The whole root system was spread out and analyzed using a scanning system (Expression 4990, Epson, CA) with a blue board as a background. Digital images of the root system were analyzed using Win RHIZO software (Régent Instruments, Québec, Canada). The total root length, total root surface area, total root volume, total projection area, and average root diameter were evaluated. The number of nodules per root was counted under a stereomicroscope.

### Determination of chlorophyll and nitrogen content

The chlorophyll in the soybean leaves was determined using samples consisting of five leaf disk obtained using a circular punch (cork borer) on five different leaves. The chlorophyll content was determined using a SPAD-502 meter (Konica-Minolta, Japan).

The roots and shoots were oven dried at 75°C for 2 days and ground. The total nitrogen content in the roots and shoots was determined using the Kjeldahl method with a nitrogen analyser (Kjeltec 2300 Autoanalyser, Foss Tecator AB, Hoganas, Sweden).

### Colonization of root tips by *bradyrhizobium japonicum*

Root colonization by *B. japonicum* under different nutrient combinations was determined under gnotobiotic conditions (25 mm in diameter, 200 mm in length). Soybean seedlings were grown in a sterilized mixture of washed sand and vermiculite (1:1). The sterilized Hoagland solutions were added as plant nutrients. The combinations of N, P, and Mg were prepared as described above: (i) HNHP, (ii) HNHMg, (iii) HNLP, (iv) HNLMg, (v) LNHP, (vi) LNHMg, (vii) LNLP, (viii) LNLMg (Table 1). The *B. japonicum* bacterial inoculant was grown overnight in YEM broth, for 4 days, and the cells were washed with 1 ml phosphate buffered saline (PBS, 20 mM sodium phosphate, 150 mM NaCl, pH 7.4) and suspended in PBS corresponding to a cell density of 10^7^ cells/ml. The germinated seeds were shaken in the bacterial suspension and sown in sterile glass tubes, one seed per tube, with 10 replicates. The tubes were kept in a plant growth chamber with a 16-h light period at 24°C and an 8-h dark period at 18°C for 10 days. At harvest, the plants were removed, and the 1-cm lengths of the root tips were shaken with PBS and plated onto YEM agar medium. The petri plates were kept at 28°C for 4–5 days, and the colonies were quantified as the CFUs per 1 cm of root tip (Simons et al., 1996).

### Statistical methods

Data on root and shoot growth, root architecture, and nitrogen and chlorophyll content were subjected to analysis of variance (ANOVA) using SPSS software (version 15). The high-range statistical domain (HSD) was used for comparison by means of Tukey's test, while means were separated using the least significant difference (LSD) test at *P* < 0.05.

## Results

### Soybean growth

The interactive effects of the different N, P, and Mg concentrations and their combinations on root and shoot fresh weight and the chlorophyll concentration in soybean leaves are reported in Table 2. Increased fresh and dry root and shoot weight and chlorophyll content in the soybean leaves were observed under a high N supply and high and low Mg concentrations in the medium (Table 2). The root dry weights were 58 and 6% higher in the HNHMg and HNLMg treatments, respectively, compared to those in the HNHP and HNLP treatments. Similarly, shoots were 47 and 14% taller in the HNHMg and HNLMg treatments, respectively, than those in the HNHP and HNLP treatments. The root and shoot dry weight was lower under low N combined either with high or low P or Mg supply in comparison to the high N combinations in all cases. No differences in plant growth parameters between P levels were observed with either low or high N supply in the medium. However, plants grown under both P levels combined with high N supply showed higher biomass as compared to low N supply (Table 2).

**Table 2:** Fresh and dry weights of roots and shoots and leaf chlorophyll concentrations of soybean inoculated with *Bradyrhizobium japonicum* USDA110.

Treatments^*^	**Root fresh weight, g/plant**	**Shoot fresh weight, g/plant**	**Root dry weight, g/plant**	**Shoot dry weight, g/plant**	**Chlorophyll content, SPAD units**
HNHP	6.1 ± 0.7bc	32.5 ± 5.7b	0.55 ± 0.09bcd	6.51 ± 0.56c	40.0 ± 1.41ab
HNHMg	10.0 ± 1.2a	41.8 ± 5.6a	0.86 ± 0.14a	9.63 ± 1.17a	42.5 ± 1.36a
HNLP	7.3 ± 0.8bc	28.8 ± 2.9b	0.60 ± 0.12bc	7.36 ± 0.48bc	35.3 ± 3.28cd
HNLMg	7.8 ± 0.7b	33.3 ± 6.3b	0.64 ± 0.06b	8.44 ± 1.08ab	37.3 ± 3.14bc
LNHP	4.9 ± 0.5cd	13.6 ± 1.4c	0.40 ± 0.07de	3.02 ± 0.50d	35.3 ± 2.70bc
LNHMg	6.4 ± 1.5bc	19.2 ± 0.9c	0.46 ± 0.09e	3.97 ± 0.56d	37.3 ± 2.74bc
LNLP	5.5 ± 1.0cd	13.1 ± 1.3c	0.32 ± 0.07cde	2.92 ± 0.54d	32.4 ± 1.63e
LNLMg	4.3 ± 1.5cd	16.5 ± 0.9c	0.35 ± 0.09e	3.13 ± 0.60d	36.0 ± 1.17cde

*Plants were grown for 45 days under hydroponic conditions with nutrient solutions supplemented with combinations of high or low N, P, and Mg concentrations*.

* *high N and high P (HNHP), high N and high Mg (HNHMg), high N and low P (HNLP), high N and low Mg (HNLMg), low N and high P (LNHP), low N and high Mg (LNHMg), low N and low P (LNLP), and low N and low Mg (LNLMg). Columns represent means for six plants (N = 6) with standard error bars, and different letters indicate significant differences between treatments at P < 0.05 (Tukey's t-test)*.

These results indicate that, regardless of the concentration, Mg promotes plant growth and development in combination with high nitrogen in the growth medium. Reducing the level of N in the nutrient solution decreased root and shoot growth under the high or low P and Mg concentrations. The chlorophyll concentration in the soybean leaves was not affected by the interactions among N, P, and Mg, although a slight increase in chlorophyll was observed under the high N supply combined with high P and high Mg concentrations (HNHP, HNHMg) (Table 2).

### Nodule number, N uptake and root colonization in *B. japonicum*

The response of the symbiotic performance of *B. japonicum* USDA110 and the host plant to the nutrient supply was assessed based on the nodule number and nodule weight (Table 3). The nodulation of soybean was inhibited by higher N supply combined with either high or low P concentrations. The nodule weight increased under low N supply in combination with both high and low P concentrations as compared to high N supply.

**Table 3:** Nodule number, nodule fresh, and dry weight, N content of soybean root and shoot inoculated with *Bradyrhizobium japonicum* USDA110.

Treatments^*^	**Nodule number per plant**	**Nodule fresh weight, g/plant**	**Nodule dry weight, g/plant**	**N content, shoot, mg/g plant**	**N content, root, mg/g plant**
HNHP	136.5 ± 45.3d	0.95 ± 0.2c	0.15 ± 0.04d	3.56 ± 0.48b	2.67 ± 0.13bc
HNHMg	186.5 ± 41.0ab	1.40 ± 0.4b	0.38 ± 0.10ab	4.20 ± 0.17a	3.44 ± 0.45a
HNLP	88.7 ± 13.2de	1.10 ± 0.3c	0.21 ± 0.04cd	2.46 ± 0.31de	2.75 ± 0.17bc
HNLMg	154.0 ± 10.8cd	1.12 ± 0.1c	0.21 ± 0.05cd	2.69 ± 0.26cd	2.85 ± 0.09b
LNHP	168.3 ± 18.8b	1.19 ± 0.1c	0.25 ± 0.04cd	2.02 ± 0.13e	2.32 ± 0.28c
LNHMg	189.0 ± 9.7a	1.72 ± 0.3a	0.39 ± 0.06a	3.50 ± 0.21b	2.82 ± 0.33b
LNLP	72.3 ± 8.3e	1.30 ± 0.2bc	0.26 ± 0.07c	2.37 ± 0.20de	2.37 ± 0.21c
LNLMg	101.7 ± 5.6de	1.20 ± 0.3bc	0.29 ± 0.06bc	2.93 ± 0.28c	2.54 ± 0.31bc

*Plants were grown for 45 days under hydroponic conditions with nutrient solutions supplemented with combinations of high or low N, P, and Mg concentrations*.

* *high N and high P (HNHP), high N and high Mg (HNHMg), high N and low P (HNLP), high N and low Mg (HNLMg), low N and high P (LNHP), low N and high Mg (LNHMg), low N and low P (LNLP), and low N and low Mg (LNLMg). Columns represent means for six plants (N = 6) with standard error bars, and different letters indicate significant differences between treatments at P < 0.05 (Tukey's t-test)*.

It appears that the number of nodules in the soybean roots increased significantly by 36 and 75% under the high N supply combined with high Mg (HNHMg) and low Mg (HNLMg) compared to both high and low P concentrations in solution. Similar observations were recorded for plants grown with a low N supply, in which the number of nodules was higher by 12 and 39% under the LNHMg and LNLMg treatment compared to soybean roots grown under low N combined with both high and low P, respectively (Table 3). In general, the nodule dry weight was higher in plants grown under low N combined either with P or Mg in comparison to the high N combinations in all cases except the HNHMg treatment. A significantly higher nodule dry weight was observed in the HNHMg and LNHMg treatments than in the other treatments.

We observed that the N content in the roots and shoots of soybean plants was significantly lower under LNHP and LNLP but that it was higher under HNHMg and LNHMg, indicating that Mg promotes N acquisition by the plant tissues (Table 3). The N concentration in the roots of plants grown under HNHMg and LNHMg increased by 17 and 73%, respectively, while the N in the shoots increased by 32 and 4%, respectively, compared to the shoots in the HNHP and LNHP treatments. N content of shoots was higher in plants grown under high P supply combined with high N supply as compared to low P supply combined with high N supply, but under low N supply, no impact of P was observed. All other treatments resulted in lower concentrations of N in the roots and shoots compared to the N accumulation in the HNHMg and LNHMg treatments.

The combinations of N, P, and Mg had different effects on the colonization of *B. japonicum* USDA110 in the rhizosphere of soybean (Table 4). Compared to the growth medium with low or high N combined with either low or high P, the N combinations with low or high Mg increased the CFU counts of *B. japonicum* USDA110. The number of rhizobial cells colonizing the soybean roots was higher under the HNHMg treatment (6.78 × 10^4^ CFUs/cm of root tip), followed by the HNLMg (4.72 × 10^4^ CFUs/cm of root tip) and LNHMg (4.10 × 10^4^ CFU/cm of root tip) conditions. The root colonization of soybean by *B. japonicum* under LNHP, LNLP, and LNLMg did not significantly differ but was lower, at 2.01 ± 0.30 × 10^4^, 1.89 ± 0.31 × 10^4^, and 1.89 ± 0.25 × 10^4^ CFUs/cm root tip (Table 4).

**Table 4:** Impacts of N, P, and Mg combinations on colonization densities of *Bradyrhizobium japonicum* and on the growth of soybean seedlings in a gnotobiotic sand-vermiculite system.

Treatments^*^	**Root length^a^**	**Shoot height^a^**	**Fresh weight^b^**	**Dry weight^b^**	**10^4^CFU/cm root**
HNHP	13.5 ± 1.00bcd	20.0 ± 1.82a	1.14 ± 0.12ab	0.14 ± 0.04ab	2.62 ± 0.56cd
HNHMg	16.0 ± 1.50ab	15.5 ± 1.00b	1.31 ± 0.23ab	0.19 ± 0.08a	6.78 ± 0.78a
HNLP	14.5 ± 1.71bcd	17.5 ± 1.29b	1.26 ± 0.15a	0.13 ± 0.01ab	3.05 ± 0.49c
HNLMg	16.2 ± 0.95a	20.5 ± 1.804	1.30 ± 0.23a	0.13 ± 0.03ab	4.72 ± 0.73b
LNHP	13.2 ± 1.50bcd	18.5 ± 0.57ab	1.12 ± 0.16ab	0.09 ± 0.03b	2.01 ± 0.30e
LNHMg	15.5 ± 1.00abc	20.0 ± 1.29a	1.12 ± 0.08ab	0.13 ± 0.05ab	4.10 ± 0.59b
LNLP	13.0 ± 1.71cd	16.0 ± 0.50b	0.95 ± 0.14b	0.09 ± 0.02b	1.89 ± 0.31e
LNLMg	11.7 ± 1.00d	17.2 ± 0.45b	1.36 ± 0.18a	0.11 ± 0.01b	1.84 ± 0.25e

Plants were grown for 12 days;

a *cm*,

b *g/plant; Different letters indicate significant differences between treatments at P < 0.05 (Tukey's t-test)*.

* *high N and high P (HNHP), high N and high Mg (HNHMg), high N and low P (HNLP), high N and low Mg (HNLMg), low N and high P (LNHP), low N and high Mg (LNHMg), low N and low P (LNLP), and low N and low Mg (LNLMg). Columns represent means for six plants (N = 6) with standard error bars, and different letters indicate significant differences between treatments at P < 0.05 (Tukey's t-test)*.

### Soybean root architecture

**Figure 1:**
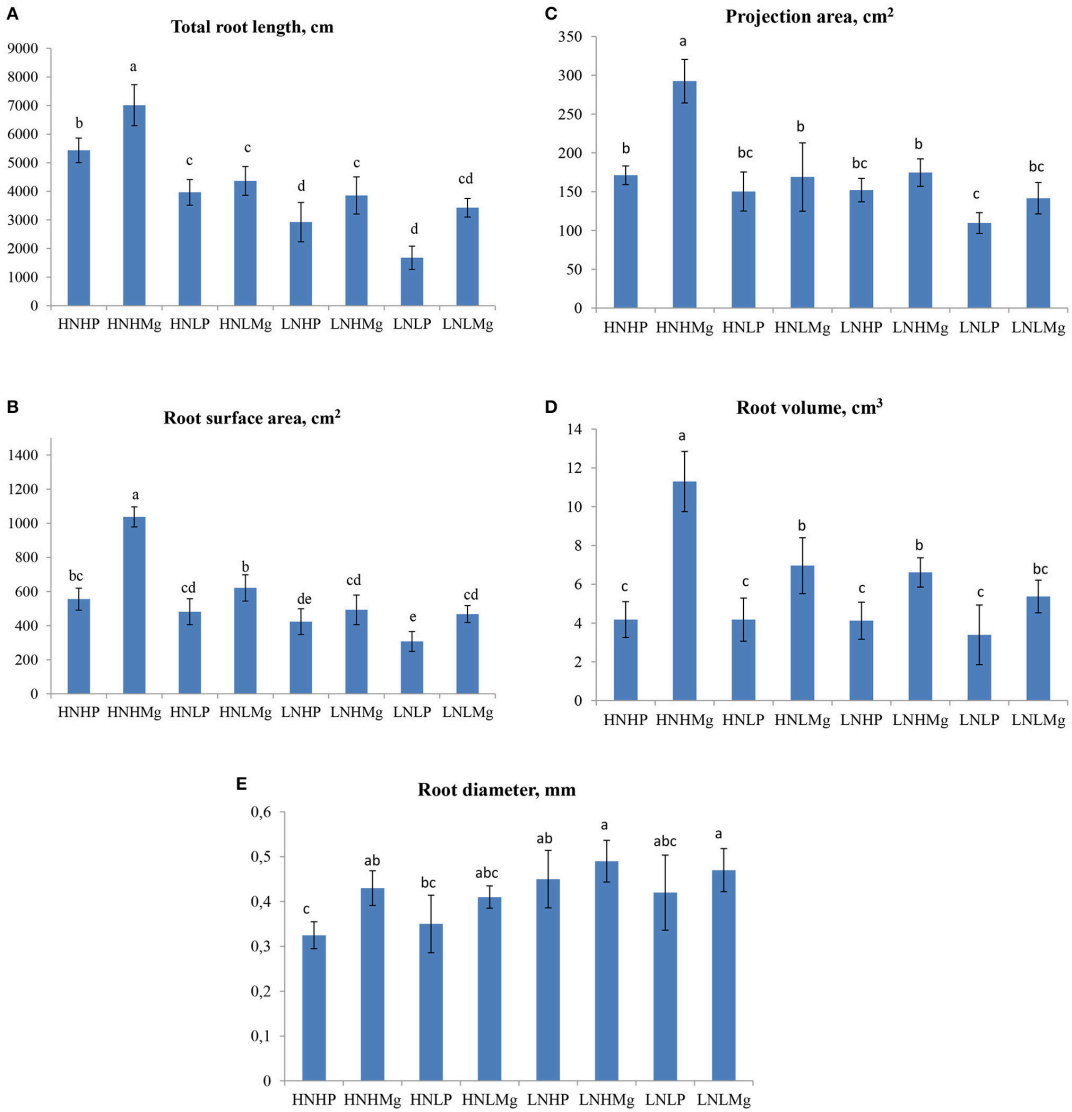
Root morphological traits [total root length **(A)**, surface area **(B)**, projection area **(C)**, root volume **(D)**, and root diameter **(E)**] of soybeans inoculated with *Bradyrhizobium japonicum* USDA110 grown under different N, P, and Mg supplies (HNHP (N−3,000 μmol/L and P−250 μmol/L), HNHMg (N−3,000 μmol/L and Mg−1,000 μmol/L), HNLP (N−3,000 μmol/L and P−50 μmol/L), HNLMg (HNHMg (N−3,000 μmol/L and Mg−250 μmol/L), LNHP (N−300 μmol/L and P−250 μmol/L), LNHMg (N−300 μmol/L and Mg−1,000 μmol/L), LNLP (N−300 μmol/L and P−50 μmol/L), LNLMg (N−300 μmol/L and Mg−250 μmol/L). Plants were grown for 45 days under hydroponic conditions. Columns represent means for five plants (*N* = 5), with standard error bars, and different letters indicate significant differences between treatments at *P* < 0.05 (Tukey's *t*-test).

In general, the soybean root architecture showed a differential response to the various nutrient treatments tested in this experiment. Interestingly, the total root length (RL) significantly increased by 5,434.0 cm/plant under HNHP and 7,012.9 cm/plant under HNHMg compared to the other treatments, indicating that high N, P, and Mg levels strongly stimulate root system growth (Figure 1A). The plants grown under LNLP showed the lowest root length, revealing nutrient deficiency effects. Mg availability changed the root surface area, especially when the plants were grown at high Mg concentrations (Figure 1B). By comparison, the surface area (SA) of the roots was 86, 115, and 66% higher in the HNHMg treatment than in the HNHP, HNLP, and HNLMg treatments. The projection area (PA) and root volume (RV) similarly responded to mineral nutrients, as they were higher under both HNHMg and HNLMg, as well as LNHMg, compared to the other treatments (Figures 1C,D). As shown in Figure 1E, the root diameter responded differently to all treatments. Interestingly, both LNHMg and LNLMg induced up to 53% larger root diameters in comparison to the other treatments. In contrast, both the HNHP and HNLP treatments resulted in a smaller root diameter.

Strong and significant positive correlations were recorded between the number of nodules, the N concentrations in the roots and shoot and the root architecture of soybean plants inoculated with *B. japonicum* USDA110 grown with the different tested nutrient combinations. Nodule number showed significant positive correlations with the average root diameter (*r* = 0.82) and N content in the root tissue (*r* = 0.78) under HNHP. Under HNHMg, the root surface area showed strong and significant positive correlations with the root projection area (*r* = 0.69), root volume (*r* = 0.91), average root diameter (*r* = 0.69), and nodule number (*r* = 0.73) (Table 5). The number of nodules in plants grown under LNHP was significantly correlated with the root surface area (*r* = 0.91) and root projection area (*r* = 0.78). Moreover, the N content of the root tissue was also correlated with the nodule number (*r* = 0.81) under LNHMg (Table 6). There were also significant correlations between nodule number and root surface area (*r* = 0.88), root projection area (*r* = 0.89), and root volume (*r* = 0.78) under the HNLP conditions (Table 7).

**Table 5:** Correlations of root length (RL), surface area (SA), projection area (PA), root volume (V), root diameter (AD), nodule number (NN), nodule weight (NdW), and nitrogen content of the shoot (NS) and root (NR) of soybean inoculated with *Bradyrhizobium japonicum* USDA110 in hydroponic culture with high nitrogen, phosphorous, and magnesium supplies (HNHP (N−3,000 μmol/L and P−250 μmol/L) and HNHMg (N−3,000 μmol/L and Mg−1,000 μmol/L).

**Parameter**	**Treatments**	**RL**	**SA**	**PA**	**V**	**AD**	**NN**	**NdW**	**NS**
SA	HNHP	0.53^**^	1.00						
	HNHMg	0.35^**^							
PA	HNHP	0.11^*^	0.48^**^	1.00					
	HNHMg	0.64^**^	0.69^***^						
V	HNHP	−0.17^*^	0.60^**^	0.77^***^	1.00				
	HNHMg	0.22^*^	0.91^***^	0.76^***^					
AD	HNHP	−0.31^*^	0.63^**^	0.55^**^	0.86^***^	1.00			
	HNHMg	0.49^**^	0.69^***^	0.98^***^	0.81^***^				
NN	HNHP	−0.22^*^	0.58^**^	0.15^*^	0.52^**^	0.82^***^	1.00		
	HNHMg	0.09	0.73^***^	0.12^*^	0.45^**^	0.09			
NdW	HNHP	0.02	0.06	−0.79^***^	−0.47^**^	−0.07	0.30^*^	1.00	
	HNHMg	0.84^***^	−0.01	0.20^*^	−0.19^*^	0.00	0.02		
NS	HNHP	−0.15^*^	−0.25^*^	0.05	−0.17^*^	−0.13^*^	−0.48^**^	−0.01	1.00
	HNHMg	−0.38^**^	−0.53^**^	−0.43^**^	−0.63^**^	−0.47^**^	−0.05	−0.08	
NR	HNHP	0.36^**^	0.78^***^	0.08	0.32^*^	0.47^**^	0.38^**^	0.46^**^	0.21^*^
	HNHMg	0.24^*^	0.14^*^	−0.02	−0.14^*^	−0.01	0.34^**^	0.04	−0.02

Significance levels for positive correlations are

* *P < 0.05 (significant)*,

** P < 0.01 (highly significant), and

*** *P < 0.001 (extremely significant)*.

**Table 6:** Correlations of root length (RL), surface area (SA), projection area (PA), root volume (V), root diameter (AD), nodule number (NN), nodule weight (NdW), and nitrogen content of the shoot (NS) and root (NR) of soybean inoculated with *Bradyrhizobium japonicum* USDA110 in hydroponic culture with low nitrogen but high phosphorous and magnesium supplies (LNHP (N−300 μmol/L and P−250 μmol/L) and LNHMg (N−300 μmol/L and Mg−1,000 μmol/L).

**Parameter**	**Treatments**	**RL**	**SA**	**PA**	**V**	**AD**	**NN**	**NdW**	**NS**
SA	LNHP	0.04	1.00						
	LNHMg	0.58^**^							
PA	LNHP	−0.62^**^	0.55^**^	1.00					
	LNHMg	0.74^***^	0.69^***^						
V	LNHP	0.21^*^	0.74^***^	0.19^*^	1.00				
	LNHMg	0.75^***^	0.26^*^	0.64^**^					
AD	LNHP	0.43^**^	0.43^**^	−0.18^*^	0.82^***^	1.00			
	LNHMg	−0.37^**^	−0.64^**^	−0.42^**^	0.24^*^				
NN	LNHP	−0.06	0.91^***^	0.78^***^	0.57^**^	0.25^*^	1.00		
	LNHMg	0.07	0.17^*^	0.59^**^	0.20^*^	0.03			
NdW	LNHP	−0.07	−0.53	−0.26^*^	−0.31^*^	0.23^*^	−0.42^**^	1.00	
	LNHMg	0.06	−0.39^**^	−0.17^*^	0.00	0.32^*^	0.31^*^		
NS	LNHP	−0.20^*^	−0.90^***^	−0.58^**^	−0.69^***^	−0.49^**^	−0.95^***^	0.33^*^	1.00
	LNHMg	0.34^**^	−0.29^*^	0.37^**^	0.55^**^	0.08	0.08	−0.15^*^	
NR	LNHP	0.46^**^	−0.09	0.01	−0.47^**^	−0.42^**^	0.14^*^	−0.12^*^	−0.16^*^
	LNHMg	−0.53^**^	−0.14^*^	0.08	−0.29^*^	0.22^*^	0.81^***^	0.19^*^	−0.17^*^

Significance levels for positive correlations are

* *P < 0.05 (significant)*,

** P < 0.01 (highly significant), and

*** *P < 0.001 (extremely significant)*.

**Table 7:** Correlations of root length (RL), surface area (SA), projection area (PA), root volume (V), root diameter (AD), nodule number (NN), nodule weight (NdW), and nitrogen content of the shoot (NS) and root (NR) of soybean inoculated with *Bradyrhizobium japonicum* USDA110 in hydroponic culture with high nitrogen, low phosphorous and low magnesium supplies (HNLP (N−3,000 μmol/L and P−50 μmol/L) and HNLMg (N−3,000 μmol/L and Mg−250 μmol/L).

**Parameter**	**Treatments**	**RL**	**SA**	**PA**	**V**	**AD**	**NN**	**NdW**	**NS**
SA	HNLP	0.33^*^	1.00						
	HNLMg	0.66^**^							
PA	HNLP	0.31^*^	0.99^***^	1.00					
	HNLMg	0.78^***^	0.91^***^						
V	HNLP	0.40^**^	0.75^***^	0.73^***^	1.00				
	HNLMg	0.44^**^	0.90^***^	0.90^***^					
AD	HNLP	−0.02	0.12^*^	0.10^*^	0.65^**^	1.00			
	HNLMg	−0.39^**^	−0.41^**^	−0.58^**^	−0.62^**^				
NN	HNLP	0.04	0.88^***^	0.89^***^	0.78^***^	0.23^*^	1.00		
	HNLMg	0.47^**^	0.39^**^	0.37^**^	0.16^*^	0.09			
NdW	HNLP	−0.39^**^	−0.48^**^	−0.60^**^	−0.26^*^	0.29^*^	−0.44^**^	1.00	
	HNLMg	0.22^*^	0.00	−0.02	−0.03	−0.54^**^	−0.43^**^		
NS	HNLP	−0.19^*^	−0.62^**^	−0.67^***^	−0.79^***^	−0.29^*^	−0.85^***^	0.55^**^	1.00
	HNLMg	−0.11^*^	−0.49^**^	−0.55^**^	−0.65^**^	0.38^**^	−0.58^**^	0.49^**^	
NR	HNLP	0.64^**^	0.65^**^	0.55^**^	0.39^**^	−0.15^*^	0.32^*^	−0.03	−0.06
	HNLMg	−0.44^**^	−0.57^**^	−0.36^**^	−0.23^*^	−0.26^*^	−0.84^***^	0.28^*^	0.33^*^

Significance levels for positive correlations are

* *P < 0.05 (significant)*,

** P < 0.01 (highly significant), and

*** *P < 0.001 (extremely significant)*.

## Discussion

**Figure 2:**
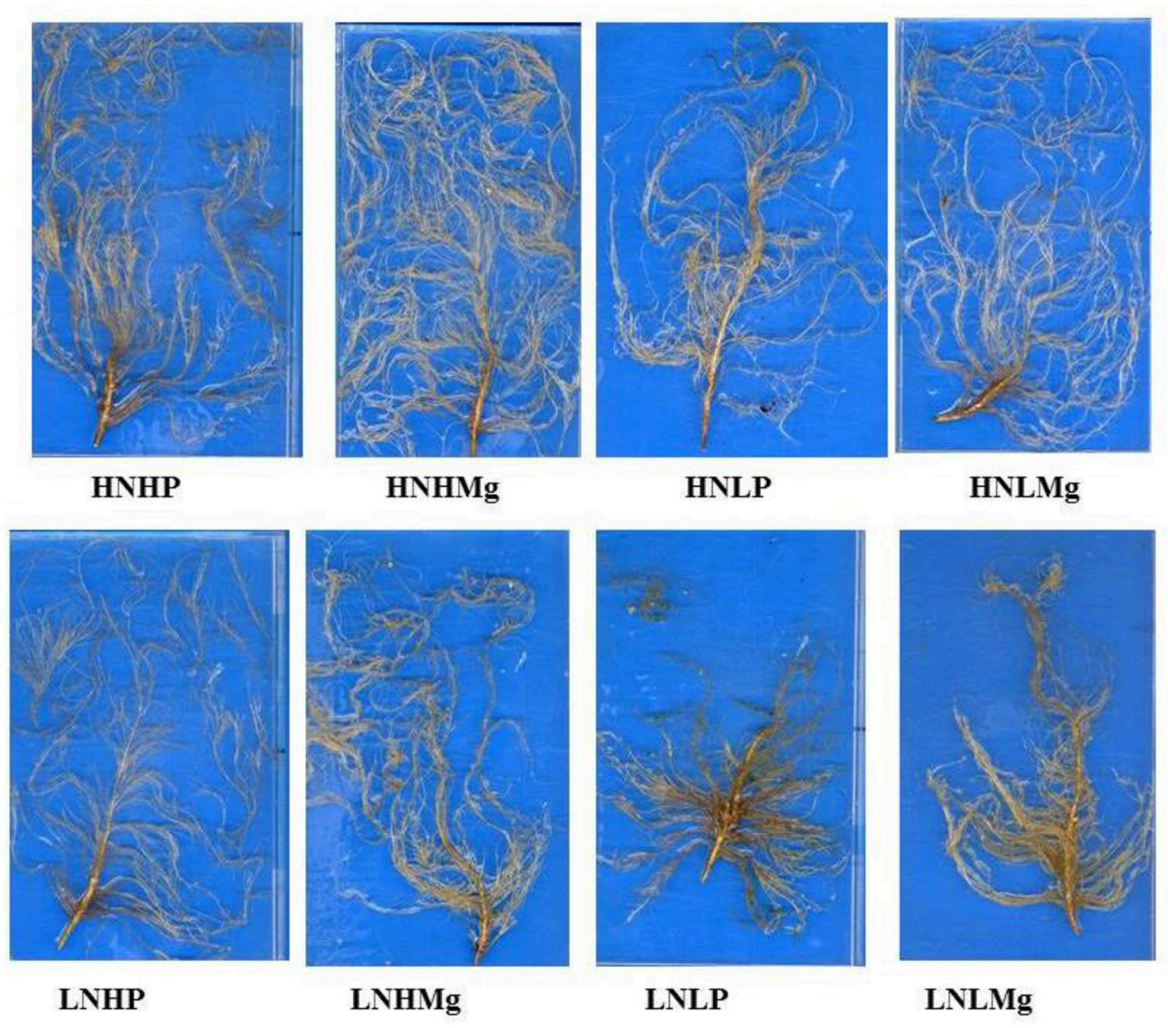
Root systems of soybean grown in Hoagland solution supplemented with different N, P, and Mg supplies under hydroponic conditions. Nutrient combinations: HNHP (N−3,000 μmol/L and P−250 μmol/L), HNHMg (N−3,000 μmol/L and Mg−1,000 μmol/L), HNLP (N−3,000 μmol/L and P−50 μmol/L), HNLMg (HNHMg (N−3,000 μmol/L and Mg−250 μmol/L), LNHP (N−300 μmol/L and P−250 μmol/L), LNHMg (N−300 μmol/L and Mg−1,000 μmol/L), LNLP (N−300 μmol/L and P−50 μmol/L), LNLMg (N−300 μmol/L and Mg−250 μmol/L).

The present study clearly showed that nutrient interactions among nitrogen, phosphorous, and magnesium have an important effect on the performance of the symbiosis between inoculated *B. japonicum* USDA110 and soybean host plants (genotype YC03-3) as well as on the development of the root system (Figure 2). In the present study, low nodulation of soybean was observed under high N supply combined with either high or low P concentrations. In earlier studies, Day et al. (1989) reported a higher nodule number of soybean grown under limited KNO_3_ (0.5 mM) supply, whereas an increased concentration of KNO_3_ (7.5 mM) inhibited nodulation. We have observed that the interaction between N and P influenced plant growth and the symbiotic performance of *B. japonicum* and soybean. The number of nodules was higher under a high P supply combined with N than low available P. This finding is supported by previous studies and was related mainly to the interaction between the supplies of N and P (Hellsten and Huss-Danell, 2000). In the present study, the lowest number of nodules was observed on soybean roots grown under a low P supply regardless of the N supply level. It has been previously reported that P deficiency limits nodule formation in leguminous plants (Kamara et al., 2008). Conversely, Bashir et al. (2011) noted that adequate P availability improves the nodule number and N content in the tissues of mung bean. Such positive effects of a high P supply on nodule development are associated with the essential function of P in energy metabolism (Tang et al., 2001). On the other hand, the adverse effects of P deficiency are likely related to the inhibition of energy-dependent reactions in the nodule cells and were associated with decreased bacteroid biomass (Sa and Israel, 1991). Thus, P deficiency may limit plant root development by affecting nodule development and function (O'Hara, 2001).

Moreover, higher N applications may alter the symbiotic performance of legumes and may cause the development of less-mutualistic rhizobia (Murray et al., 2017). Dean et al. (2014) also demonstrated the inhibition of nodule formation by *Bradyrhizobium* in soybean by increasing the N concentration in the soil. Notably, there were no significant differences in shoot biomass under a high or low P supply. This observation is in agreement with the findings of Gentili and Huss-Danell (2003). The root and shoot biomass of soybean treated with *B. japonicum* were higher under high N in combination with high Mg in comparison to low Mg, indicating a synergistic effect of these nutrients. In contrast, under a low nitrogen supply, both high and low concentrations of Mg facilitated plant growth and root system development, although there were no significant differences when compared to the previously described treatment.

The positive effect of Mg on root development has been reported in many studies, e.g., higher root and shoot biomass were observed in banana grown under an increased concentration of Mg (370 mg L^−1^ of MgSO_4_) in comparison to a lower concentration in the growth medium (185 mg L^−1^ of MgSO_4_) (Rodrigues et al., 2017). A similar observation was reported by Chen et al. (2017), who found that a higher Mg supply significantly improved root and shoot biomass and N and P uptake in soybean through the improved symbiotic performance of arbuscular mycorrhizal fungi (AMF) and rhizobia, both of which showed statistically significant positive correlations with the growth of the root system. In another study, the root growth of *Arabidopsis* was inhibited by low Mg and P supplies but was improved under a high P supply in the growth solution (Niu et al., 2015).

Notably, improved N uptake by roots could be observed in soybean grown in media supplemented with Mg compared to media supplemented with P. It has been documented that N acquisition by plants not only depends on the available nitrogen forms but also on the interactions of N with other nutrients (Leidi and Rodríguez-Navarro, 2000), such as Mg in the present study. Furthermore, Grzebisz (2013), in his extensive review on magnesium fertilization, observed that adding Mg to the plant growth medium affected nitrogen acquisition and described it as “magnesium-induced nitrogen uptake.”

In an earlier report, it was observed that the composition of media has strong effects on bacterial colonization and biofilm formation (McEldowney and Fletcher, 1986). For example, a high nitrogen concentration inhibited root colonization of sugarcane by *Acetobacter diazotrophicus* (Fuentes-Ramírez et al., 1999), whereas Mg increased colonization and biofilm formation by *Pseudomonas fluorescens* (Song and Leff, 2006). The colonization capacity of rhizobia in the rhizosphere is crucial for the successful association with host plants and formation of mutualistic relationships, and it is strongly affected by the available nutrients in the plant growth media (Kuzyakov and Xu, 2013). In our study, higher rhizosphere colonization by *B. japonicum* was observed in plants grown with nutrient combinations containing Mg. In previous studies, Vincent et al. (1962) reported the failure of rhizobial cell division in Mg-deficient growth media. Conversely, Kiss et al. (2004) observed a higher number of and larger nodules in pea roots treated with Mg in comparison to untreated roots. These findings indicate the importance of Mg in nodule formation and rhizobium activity in the roots, such as proliferation and nitrogen fixation.

Plant roots are known to be sensitive to the nutrient supply; furthermore, external nutrient availability can alter root morphology (Giehl et al., 2014; Li et al., 2016). In the present study, we found a strong interaction between N and Mg, which affected the root morphology of soybean inoculated with *B. japonicum* USDA110. Root morphology responded to the P concentration and was stimulated under high P but declined under a low P supply combined with high or low N. A similar observation was reported by López-Bucio et al. (2002), who indicated that a low P supply inhibited root growth in *Arabidopsis* compared to an adequate P supply. Liu et al. (2016) reported that the morphological and physiological parameters of white lupin and faba bean did not change under N supply (NH_4_NO_3_) and P deficiency in a hydroponic system. Similar studies have indicated that P deficiency limits the length of the main and lateral roots, the number of nodules, and the nitrogen fixation process (Liao et al., 2001). In our study, root diameter responded to nutrient supply in a similar way as nodule number, whereas N and Mg showed a positive effect. In an earlier study, Arima et al. (2000) reported that the nodule distribution in the root is closely related to the number of vascular bundles within a root diameter class. We also observed significantly positive correlations between nodule number, N content in the plant tissues and root growth parameters of soybean plants grown in the presence of Mg.

## Conclusion

Our results indicate that the symbiotic association between *B. japonicum* and soybean can be modulated by different nutrient concentrations in the growth media. The interactions among N, P, and Mg affect the growth of soybean by improving the symbiotic performance of *B. japonicum* and its root growth parameters. We observed significant positive correlations between the number of nodules, N content in the plant tissues and root growth parameters of soybean plants grown with a Mg supply combined with N, which highlights the importance of N and Mg availability in the growth medium for regulating the root system architecture and nodule formation. Neither root nor shoot growth responded significantly to P availability regardless of the N supply. The colonization of the rhizosphere by *B. japonicum* was positively affected by the Mg supply combined with N, indicating the importance of Mg in bacterial proliferation. Taken together, these findings contribute to new insights for the improvement of the root system and the symbiotic performance of rhizobia inoculated on legumes, stressing the importance of a balanced supply of nutrients.

## Author contributions

DE and SW designed the experiment. DE and DJ conducted the experiment. PA and MA analyzed the data. DE, SW, and PA wrote the manuscript. All authors read and approved the manuscript.

### Conflict of interest statement

The authors declare that the research was conducted in the absence of any commercial or financial relationships that could be construed as a potential conflict of interest.
